# Heterologous Lactate Synthesis in *Synechocystis* sp. Strain PCC 6803 Causes a Growth Condition-Dependent Carbon Sink Effect

**DOI:** 10.1128/aem.00063-22

**Published:** 2022-04-04

**Authors:** Marcel Grund, Torsten Jakob, Jörg Toepel, Andreas Schmid, Christian Wilhelm, Bruno Bühler

**Affiliations:** a Department of Solar Materials, Helmholtz Center for Environmental Research—UFZ, Leipzig, Germany; b Plant Physiology Group, Institute for Biology, University of Leipzig, Leipzig, Germany; c Department of Algal Biotechnology, Institute for Biology, University of Leipzig, Leipzig, Germany; Kyoto University

**Keywords:** carbon fixation, cyanobacteria, electron flow, lactate production, photosynthetic efficiency, quantitative physiology, source sink balance

## Abstract

Cyanobacteria are considered promising hosts for product synthesis directly from CO_2_ via photosynthetic carbon assimilation. The introduction of heterologous carbon sinks in terms of product synthesis has been reported to induce the so-called “carbon sink effect,” described as the release of unused photosynthetic capacity by the introduction of additional carbon. This effect is thought to arise from a limitation of carbon metabolism that represents a bottleneck in carbon and electron flow, thus enforcing a downregulation of photosynthetic efficiency. It is not known so far how the cellular source/sink balance under different growth conditions influences the extent of the carbon sink effect and in turn product formation from CO_2_, constituting a heterologous carbon sink. We compared the *Synechocystis* sp. strain PCC 6803 wild type (WT) with an engineered lactate-producing strain (SAA023) in defined metabolic states. Unexpectedly, high-light conditions combined with carbon limitation enabled additional carbon assimilation for lactate production without affecting biomass formation. Thus, a strong carbon sink effect only was observed under carbon and thus sink limitation, but not under high-sink conditions. We show that the carbon sink effect was accompanied by an increased rate of alternative electron flow (AEF). Thus, AEF plays a crucial role in the equilibration of source/sink imbalances, presumably via ATP/NADPH balancing. This study emphasizes that the evaluation of the biotechnological potential of cyanobacteria profits from cultivation approaches enabling the establishment of defined metabolic states and respective quantitative analytics. Factors stimulating photosynthesis and carbon fixation are discussed.

**IMPORTANCE** Previous studies reported various and differing effects of the heterologous production of carbon-based molecules on photosynthetic and growth efficiency of cyanobacteria. The typically applied cultivation in batch mode, with continuously changing growth conditions, however, precludes a clear differentiation between the impact of cultivation conditions on cell physiology and effects related to the specific nature of the product and its synthesis pathway. In this study, we employed a continuous cultivation system to maintain defined source/sink conditions and thus metabolic states. This allowed a systematic and quantitative analysis of the effect of NADPH-consuming lactate production on photosynthetic and growth efficiency. This approach enables a realistic evaluation of the biotechnological potential of engineered cyanobacterial strains. For example, the quantum requirement for carbon production was found to constitute an excellent indicator of the source/sink balance and thus a key parameter for photobioprocess optimization. Such knowledge is fundamental for rational and efficient strain and process development.

## INTRODUCTION

Similar to plants, cyanobacteria are capable of utilizing CO_2_ and light as sources for carbon and energy, respectively, and can be genetically engineered for the production of carbon-based products from CO_2_, which gives them the potential to contribute to a sustainable and fossil resource-free bioeconomy. In comparison to crop plants, cyanobacteria possess distinct advantages for biotechnological applications, such as faster growth, higher annual area-based carbon fixation rates (1), genetic accessibility (2, 3), and reduced competition with agricultural land use (4).

Cyanobacteria have been engineered to synthesize a variety of bioproducts from CO_2_ (reviewed in references 5 to 7): e.g., hydrocarbons (8–11), fatty acids (12), organic acids (13–15), and alcohols (16, 17), as well as sugars (18, 19). These products have in common that their synthesis requires CO_2_ fixation via the Calvin-Benson-Bassham (CBB) cycle and may need additional NADPH and/or ATP from the photosynthetic light reaction. Since the capacity of the light reaction (source) exceeds the sink capacity of carbon fixation by far (20), the CBB cycle with its specific ATP and NADPH demands represents a bottleneck in the metabolism and is responsible for the so-called “carbon sink effect” (21). This effect manifests itself by an increase of the photosynthetic efficiency due to the introduction of additional/artificial electron sinks: e.g., the reduction of nitrate (22), electron withdrawal by cytochrome P450 (23), or the introduction of heterologous carbon sinks requiring additional carbon fixation and thus electrons (24, 25). With heterologous carbon sinks, some studies observed an enhanced carbon fixation rate in comparison to the respective wild-type (WT) strain (11, 16, 18, 25–28). Obviously, photosynthetic cells do not fully exploit their intrinsic metabolic potential, but have to constantly adjust the source/sink balance to changing environmental conditions in order to avoid cellular damage by excessively absorbed light energy (29).

In light of the carbon sink effect, it is not surprising that metabolic engineering and optimization of carbon partitioning to a heterologous product and of the final product titer have focused on improvement of CBB cycle efficiency (30–32). Although these approaches enabled enhanced bioproduct formation, basic questions are still not fully answered: why do cyanobacterial cells keep their intrinsic metabolic capacity well below its maximum, and what are the molecular mechanisms to balance light and dark reaction capacities?

A key element in the source/sink regulation could be the ATP/NADPH ratio. Linear photosynthetic electron flow generates ATP and NADPH in a ratio that is too low to meet the requirements of the CBB cycle (33). Cells can adjust the ATP/NADPH ratio via alternative electron flow (AEF) contributing to ATP but not to NADPH regeneration (33–36). Besides the cyclic electron flux around photosystem I (PSI), AEF also comprise noncyclic electron fluxes, where electrons from water splitting are transferred to alternative electron acceptors such as O_2_ instead of NADP^+^. With a view to the biotechnological potential, it is therefore not only important how heterologous sinks influence the source versus sink capacity, but also if they exert a positive effect on the ATP/NADPH ratio. The heterologous production of isopropanol in *Synechocystis* sp. strain PCC 6803 is exemplary for such a positive effect (28). The synthesis of isopropanol by a secondary alcohol dehydrogenase consumes one additional NADPH (per molecule of isopropanol) and results in a more than 2-fold-higher ATP/NADPH ratio and an almost doubled photosynthesis rate in the isopropanol-producing strain compared to the WT strain (28). The effects of isoprene production in Synechococcus elongatus might be explained in a similar way (9). The synthesis of one isoprene molecule consumes one ATP but two NADPHs and two additional electrons from ferredoxin. Thus, isoprene production should increase the ATP/NADPH ratio, which is in line with the observation that isoprene-producing strains increased the carbon fixation rate by up to 55% and photosynthetic electron transport by even up to 80% compared to WT cells (9).

An underscored aspect in the investigation of the carbon sink effect in cyanobacteria is the influence of growth conditions, specifically of light and CO_2_. The metabolic state and the balance of source/sink capacity strongly depend on the amount of incident irradiation and CO_2_ supply (37). A combination of low irradiance and excess CO_2_ drives the cells toward a source limitation, whereas a combination of high irradiance and low CO_2_ supply enforces the sink limitation. In the latter case, the cells are forced to downregulate the photosynthetic efficiency to avoid photodestruction, and a strong carbon sink effect is expected upon introduction of a heterologous electron or carbon sink. In contrast, the carbon sink effect is expected to be smaller or even absent in the case of a low source/sink ratio. The appearance of a carbon sink effect is usually interpreted as an indicator of a “successful” induction of heterologous electron/carbon sinks, but may become hidden by a low source/sink ratio. Further, it is not clear what benefits may be achieved via growth condition optimization and how far they involve a carbon sink effect. These aspects have not been adequately considered so far. Cyanobacteria are usually grown in batch mode, with light and nutrient (including CO_2_) availabilities continuously changing over time. This forces cells to constantly acclimate to these changing sink/source conditions by adapting their metabolic state. This complicates the evaluation of specific impacts of light and nutrient availabilities on the carbon sink effect and product yield.

This study investigates the carbon sink effect in the model cyanobacterium *Synechocystis* sp. strain PCC 6803 (from now on *Synechocystis*) in a systematic and quantitative way. A continuous cultivation approach was applied to investigate defined metabolic states and source/sink ratios for the WT and the engineered lactate-producing SAA023 strain (13). SAA023 forms lactate from pyruvate via a heterologous NAD(P)H-dependent lactate dehydrogenase. The ATP over NADPH demand of lactate formation is almost 2-fold lower than for biomass production (38), auguring well for a pronounced carbon sink effect. We address the questions of whether the source/sink ratio influences the extent of the carbon sink effect and how this effect relates to the lactate production rate.

## RESULTS AND DISCUSSION

### The metabolic cell state is constantly changing during batch cultivation, with a strong impact on lactate production efficiency.

**FIG 1:**
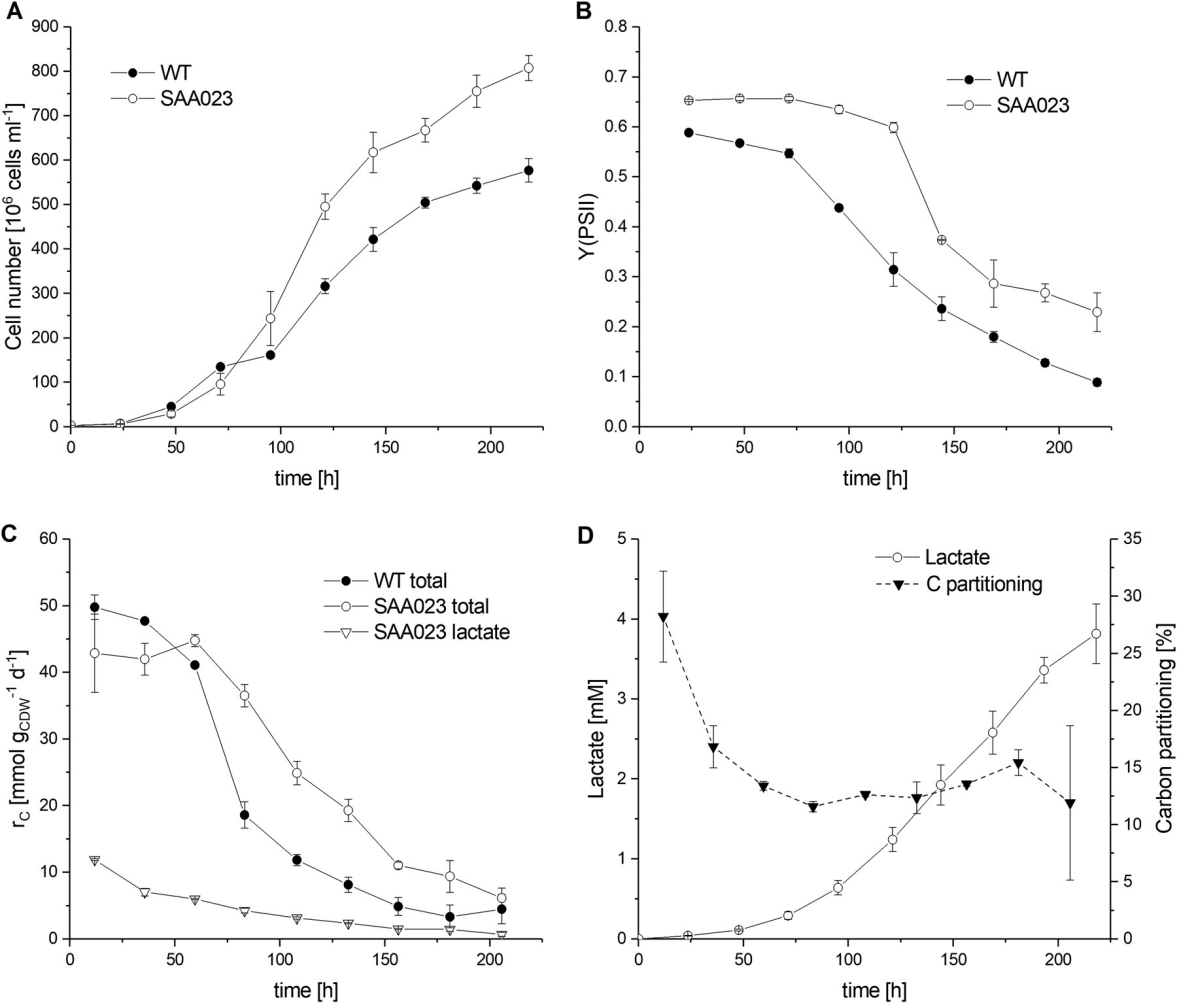
Physiological parameters of the *Synechocystis* wild type (WT) and strain SAA023 during batch cultivation (ambient CO_2_, 50 μmol photons m^−2^ s^−1^, 30°C, pH 7.8). Panels A and B show the courses of cell density and Y(PSII) (photosynthetic quantum yield at PSII). Panel C depicts total carbon production rates and the respective share used for lactate formation (SAA023 only), whereas panel D depicts the courses of the lactate concentration and of the carbon share used for lactate formation by SAA023 (carbon partitioning). Data are presented as mean values and standard deviations from duplicate experiments.

In a first step, the physiological effects of growth conditions were investigated in batch cultures (shaking flasks, ambient CO_2_) for the *Synechocystis* WT strain and a respective strain engineered for lactate production, SAA023. Due to the increasing cell numbers during batch cultivation, nutrient (including CO_2_) and light availability are constantly changing (39), with the cells forced to constantly adjust their metabolic state. For the WT strain, this metabolic acclimation could be discriminated into three phases. In phase I (0 to 75 h postinoculation), cell numbers increased exponentially, indicating unlimited growth (Fig. 1A). Cells showed a constantly high photosynthetic performance as indicated by effective quantum yield at PSII [Y(PSII)] (Fig. 1B) and carbon production rate (*r_C_*) (Fig. 1C). In the second phase (75 to 150 h), exponential growth of the WT changed into linear growth, accompanied by a severe decrease of *r_C_* and Y(PSII), indicating the onset of light and/or nutrient limitation. In the third phase (150 to 220 h), *r_C_* remained at a very low level of approximately 10% of the initial value, while Y(PSII) further decreased. This is in line with the strongly diminished growth rate of WT cells, which reached a stationary phase after 220 h. Thus, during phases II and III, WT cells are facing a strongly diminished carbon sink capacity due to a decreasing nutrient supply. The time shift in the responses of carbon production and photosynthetic performance indicate that CO_2_ limitation caused negative feedback on photosynthesis. It remains open, however, if the changing light conditions due to the increasing cell density additionally influenced the described effects.

The introduction of lactate as a heterologous carbon sink in SAA023 severely changed the physiology of the cells and the temporal appearance of the different phases of metabolic acclimation during batch cultivation. The duration of the exponential growth phase increased to 120 h postinoculation (Fig. 1A). Thereby, SAA023 showed a 12% higher photosynthetic efficiency [Y(PSII)] than the WT, with the Y(PSII) remaining high throughout this prolonged phase I. Lactate accumulation (Fig. 1D) showed a similar exponential behavior to growth (Fig. 1A). Whereas WT cultures did not accumulate detectable lactate concentrations (data not shown), SAA023 produced up to 3.8 ± 0.4 mM lactate after 220 h of cultivation. This is 5-fold higher than reported for the same strain before (13, 40), but similar to other lactate-producing strains of *Synechocystis* (13). The withdrawal of carbon by lactate production induced a sink effect in terms of a distinctly higher Y(PSII) (Fig. 1B). Interestingly, *r_C_* of SAA023 was lower during the first 40 h, correlating with slower growth compared to the WT (Fig. 1A and C). This coincided with a high carbon partitioning to lactate formation (28% ± 4% within the first 24 h) (Fig. 1D). At a later stage of batch cultivation (>150 h postinoculation), carbon production became limited and the growth rate, Y(PSII), and *r_C_* of SAA023 decreased—however, later and to a lesser extent than observed for WT cells. The carbon partitioning to lactate remained at a level of 13% and Y(PSII) at a distinctly higher value than for the WT. Furthermore, overall carbon production was higher in SAA23 than in the WT (Fig. 1C), altogether indicating a persisting carbon sink effect.

### Specific growth conditions force cells into different metabolic states.

In the later stage of the batch cultivation described above, the increasing cell concentration caused increasing light and/or nutrient limitation, enforcing a continuous metabolic source/sink balancing (41). Under these dynamic conditions, it is not possible to evaluate how and to what extent the observed physiological differences among SAA023 and WT are related to the lactate-formation-dependent carbon sink effect and how this is interconnected with the increasing nutrient limitation (42, 43). Consequently, a continuous cultivation approach, which ensures a defined and constant light and CO_2_ supply and thus enables the establishment of defined metabolic states, was chosen to investigate the physiological effect of a heterologous carbon sink (44). Cells were grown in bioreactors in turbidostat mode as described previously (22). Three different metabolic states were established and tested: (i) source limitation, i.e., low light and high carbon availability (LLHC) (1% CO_2_, 65 μmol photons m^−2^ s^−1^); (ii) sink limitation, i.e., high light and low carbon availability (HLLC) (ambient-like CO_2_ [∼0.05%], 250 μmol photons m^−2^ s^−1^); and (iii) excess in source and sink supply, i.e., high light and high carbon availability (HLHC) (1% CO_2_, 250 μmol photons m^−2^ s^−1^).

Under source limitation (LLHC), cells are expected to optimize photon uptake and photosynthetic performance. Cells then may either not be able to provide additional energy (ATP/NADPH) to support a heterologous carbon sink or may withdraw carbon and energy for lactate production at the expense of biomass formation. A pronounced carbon sink effect is not expected. Under excess conditions (HLHC), carbon fixation and biomass production rates are expected to be maximal. The higher sink capacity should allow for a higher light absorption capacity and photosynthetic efficiency compared to carbon limitation (HLLC [see below]). Two scenarios can be sketched for the effect of a heterologous carbon sink. If the cells already have reached their upper limit of carbon fixation, the heterologous synthesis of lactate would compete with biomass formation for metabolically available carbon. If carbon fixation can be further increased—e.g., due to a limitation of biomass formation by pathways other than the CBB cycle or by nutrients other than CO_2_ (43)—lactate production could be fueled by additionally fixed carbon and thus lead to a pronounced carbon sink effect. In both scenarios, a sink effect resulting in improved photosynthetic efficiency is expected. Under sink-limited conditions (HLLC), cells have to cope with excess light in relation to their sink capacity and usually adapt by reducing their light absorption capacity and increasing dissipation of excessively absorbed light energy. This is expected to result in a lower efficiency of photosynthetic light reactions and in lactate formation at the expense of biomass production due to limited carbon availability. A pronounced carbon sink effect thus rather is not expected. However, the lower ATP over NADPH demand of lactate production may help to optimize the source/sink balance and thus result in a higher overall photosynthetic performance compared to the WT.

The different physiological cell states we evaluated by determining the chlorophyll *a* (Chl*a*) content (absorption capacity), Y(PSII) (photosynthetic efficiency), and the quantum requirement per carbon (*Q_C_*) (energy requirement for total carbon production, biomass plus lactate). The comparison of these parameters showed that the chosen cultivation conditions clearly induced different physiological states of WT cells. Accordingly, source-limited cells (LLHC) possessed the highest Chl*a* content and Y(PSII) and the lowest *Q_C_* (Table 1), which met the expectation of a high photosynthetic efficiency (see above). Sink limitation (HLLC) and excess conditions (HLHC) led to 60 and 45% lower Chl*a* contents and 41 and 21% lower Y(PSII)s, respectively, indicating the expected reduction in light reaction capacity. Regarding *Q_C_*, however, HLHC did not lead to an increase, whereas HLLC resulted in a 52% higher value. Interestingly, cells were not capable of good source/sink balancing under HLLC conditions, whereas this was the case under HLLC conditions (Table 1). Obviously, the excess carbon supply under HLHC enabled the cells to adjust their metabolic capacity to the 4-fold-higher light intensity. In conclusion, the applied continuous cultivation conditions induced distinctly different metabolic states with respect to the source-to-sink capacity in the WT.

**TABLE 1:** Basic physiological parameters of *Synechocystis* WT and lactate-producing SAA023 strains under different source/sink conditions

Parameter^*a*^	Result for^*b*^:					
	Light limitation (LLHC)		Excess supply (HLHC)		Carbon limitation (HLLC)	
	WT	SAA023	WT	SAA023	WT	SAA023
*D* = μ (day^−1^)	0.82	0.67	2.41	1.86	1.13	1.20
*c*(Chl*a*) (mg Chl*a* L^−1^)	1.79 ± 0.02	2.17 ± 0.16	1.13 ± 0.14	1.28 ± 0.10	1.13 ± 0.14	1.18 ± 0.02
Chl*a*_CDW_ (mg Chl*a* g_CDW_^−1^)	19.9 ± 0.7	19.4 ± 3.0	11.0 ± 1.8	10.8 ± 1.4	8.0 ± 1.2	8.7 ± 0.5
Y(PSII)	0.56 ± 0.01	0.58 ± 0.01	0.44 ± 0.02	0.53 ± 0.01	0.33 ± 0.05	0.41 ± 0.02
a*_phy_ (m² g Chl*a*^−1^)	20.0 ± 0.3	17.7 ± 0.6	21.6 ± 2.1	20.0 ± 1.0	25.8 ± 1.4	24.6 ± 0.1
*Q*_phar_ (mol photons g_CDW_^−1^ day^−1^)	1.63 ± 0.07	1.34 ± 0.26	4.25 ± 1.13	3.55 ± 0.66	3.29 ± 0.76	3.38 ± 0.25
*Q_C_* (mol photons mol C^−1^)	50.3 ± 0.5	43.1 ± 0.4	49.9 ± 3.9	41.9 ± 4.7	76.5 ± 5.1	58.2 ± 2.2
*r_O_* (mmol O_2_ g_CDW_^−1^ day^−1^)	53.1 ± 2.2	50.0 ± 9.1	91.5 ± 16.1	91.8 ± 15.3	65.6 ± 14.4	74.8 ± 11.1
*r_F_* (mmol O_2_ g_CDW_^−1^ day^−1^)	114.8 ± 5.0	97.5 ± 17.9	232.8 ± 53.6	234.9 ± 42.8	134.4 ± 28.2	172.4 ± 19.2
*r*_Lac_ (mmol g_CDW_^−1^ day^−1^)	0.10 ± 0.01	1.62 ± 0.05	0.23 ± 0.01	5.20 ± 0.3	0.07 ± 0.01	4.29 ± 0.1
C/N (mol C mol N^−1^)	4.92 ± 0.03	5.44 ± 0.17	5.13 ± 0.08	5.42 ± 0.20	5.94 ± 0.50	5.50 ± 0.11

a *D*, dilution rate equaling the specific growth rate μ; *c*(Chl*a*), volumetric chlorophyll *a* concentration; Chl*a*_CDW_, biomass-specific Chl*a* concentration; Y(PSII), effective quantum yield at PSII; a*_phy_, Chl*a* normalized absorption coefficient of the cells; *Q*_phar_, rate of photosynthetically absorbed photons; *Q_C_*, absorbed photon requirement for total carbon production (biomass plus lactate); *r_O_*, gross O_2_ evolution rate; *r_F_*, fluorescence-based electron flux at PSII expressed as O_2_ evolution; *r*_Lac_, specific lactate formation rate; C/N, molar ratio of carbon and nitrogen in biomass.

b The tested conditions refer to low light and high carbon (LLHC), high light and high carbon (HLHC), and high light and low carbon (HLLC). Data are depicted as mean values and standard deviations from at least three replicates. See Materials and Methods for details.

### Source-limited cells do not profit from a heterologous carbon sink.

Strain SAA023 was now investigated to reveal the effect of a heterologous carbon sink under these different metabolic cell states.

**FIG 2:**
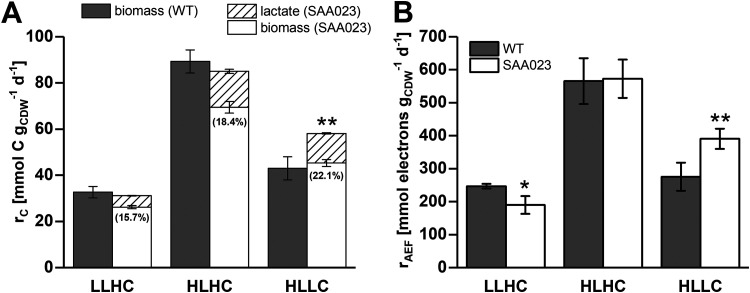
Total carbon production rates (*r_C_*) (A) and alternative electron fluxes (*r*_AEF_) (A) of *Synechocystis* WT and SAA023 strains under different growth conditions in continuously operated flat-panel photobioreactors. For strain SAA023, the carbon partitioning into lactate formation (percentage of total carbon production) is depicted by the values given in brackets. WT cells produced lactate only in trace amounts (Table 1). Cells were grown under light limitation (1% CO_2_, 65 μmol photons m^−2^ s^−1^ [LLHC]), excess condition (1% CO_2_, 250 μmol photons m^−2^ s^−1^ [HLHC]), and carbon limitation (ambient CO_2_, 250 μmol photons m^−2^ s^−1^ [HLLC]). The values represent means from measurements conducted at three different measuring days. Analysis on statistical differences between *Synechocystis* WT and SAA023 strains were performed by Student's *t* test. Data were considered significant for *P* values of <0.05 (*) and <0.01 (**).

Source-limited (LLHC) cells of SAA023 showed no increase in the total carbon production rate (biomass carbon plus lactate) (Fig. 2A) and only a minor increase of Y(PSII) (Table 1), indicating the absence of a pronounced carbon sink effect. Assimilated carbon was distributed among lactate and biomass formation, resulting in a growth rate decrease by 18% for SAA023 compared to the WT (Table 1). A lower growth rate compared to the WT also has been observed in another lactate-producing strain of *Synechocystis* sp. strain PCC 6803, SAW041, grown under LLHC conditions (45). The additional carbon sink lactate did not enhance the metabolic performance. Obviously, the WT already exploited the maximum capacity of energy supply by the light reaction. However, the decreased rate of AEF (*r*_AEF_) (Fig. 2B) indicates that lactate production influenced the ATP/NADPH ratio in cells of SAA023. AEF comprises photosynthetic water splitting-derived electrons not used for the NADP^+^ reduction, but instead channeled into alternative reactions, such as nitrate reduction, the Mehler reaction, or cyclic electron flow around PSI (37). Since these electrons still contribute to the formation of the proton gradient across the thylakoid membrane, the AEF promotes synthesis of additional ATP. The main function of AEF is the dissipation of excessively absorbed light energy. However, even under limited light conditions, AEF pathways are required to adjust the ATP/NADPH ratio by the provision of extra ATP (37). In the present study, AEF in WT cells was estimated to consume 58% of the water-derived electrons under source-limited conditions (LLHC). Notably, AEF decreased to 48% in SAA023 compared to WT cells (Fig. 2B). Since almost 16% of the carbon fixed by SAA023 was excreted as lactate (Fig. 2A), the lower ATP consumption of lactate synthesis compared to biomass production (38) constitutes a reasonable explanation for the decreased rate of AEF. Lactate production also positively influenced the photosynthetic efficiency as deduced from the lower quantum requirement of carbon production in SAA023 (43 mol photons mol C^−1^) compared to the WT (49 mol photons mol C^−1^). However, despite the positive effect of lactate formation on photosynthetic efficiency, no explicit carbon sink effect was observed under source limitation.

### Light and carbon excess enables high metabolic rates, which are not further increased by an additional carbon sink.

Light and carbon excess (HLHC) strongly increased photosynthetic performance and growth rate of WT cells (Table 1). The measured carbon production rate of 3.75 mmol C g_CDW_^−1^ h^−1^ (Fig. 2A) corresponds to the maximum calculated carbon uptake rate of *Synechocystis* (3.7 mmol C g cell dry weight [g_CDW_]^−1^ h^−1^) (37). This suggests that WT cells reached their maximum metabolic capacity under HLHC. Thereby, a high growth rate came along with a severely increased fraction of AEF (77% of electrons originating from PSII) (Fig. 2A). Given the low quantum requirement for carbon production (*Q_C_*) (Table 1), excess light energy dissipation appeared not to be the major cause for the elevated AEF (46). Instead, the high AEF may be required to achieve an adequate ATP/NADPH ratio. However, the introduction of lactate synthesis as a heterologous carbon sink in SAA023 did neither influence the total carbon production rate (biomass carbon plus lactate) (Fig. 2A) nor AEF (Fig. 2B), but again led to an increase in photosynthetic efficiency, as indicated by a higher Y(PSII) and a lower *Q_C_* (Table 1). Obviously, under light and carbon excess conditions, the carbon assimilation capacity could not be further increased via the additional carbon sink (i.e., an explicit carbon sink effect did not occur).

### Carbon limitation induced an unexpectedly strong carbon sink effect.

Under sink-limited conditions (HLLC), the introduction of lactate synthesis induced a strong improvement of photosynthetic performance, i.e., higher Y(PSII), gross O_2_ evolution rate, and fluorescence-based electron transport rate (Table 1). This positive effect on photosynthesis was an expected result considering the lower ATP over NADPH demand of lactate production. It confirms the expectation that under sink limitation, (i) cyanobacterial cells are forced to decrease light reaction capacity and (ii) a heterologous carbon sink has the potential to optimize the source/sink balance and to release unused photosynthetic capacity in *Synechocystis*. It was, however, also expected that WT cells under carbon-limited conditions already exploit their maximum carbon assimilation capacity and that a heterologous sink should compete for carbon at the expense of biomass formation. This was surprisingly not the case, and the total carbon production rate increased by almost 35% in SAA023 compared to the WT (Fig. 2A), accompanied by a strong decrease of *Q_C_* (Table 1) and a 25% increase of AEF (Fig. 2B). These results indicate that the source/sink balance in the WT under HLLC was quite far from optimal and that the introduction of a heterologous carbon sink in SAA023 strongly improved this balance. This is corroborated by the carbon sink effect observed with cells cultivated in batch mode (Fig. 1), which presumably were carbon limited at later stages of cultivation at the ambient CO_2_ concentration. Further, this is in agreement with a study on the effect of NADPH-consuming isopropanol production in *Synechocystis* at low carbon supply, reporting a carbon assimilation rate increase by almost 40% and a more than 2-fold intracellular ATP/NADPH ratio in the producer strain compared to the WT (28). Concomitantly, the isopropanol-producing strain showed a distinctly higher electron transport rate in PSI, indicating an increased AEF. Considering the behavior of *Q_C_* in the investigated system, it obviously constitutes an excellent indicator for the source/sink balance and thus can be used as key parameter to support the optimization of growth conditions.

**FIG 3:**
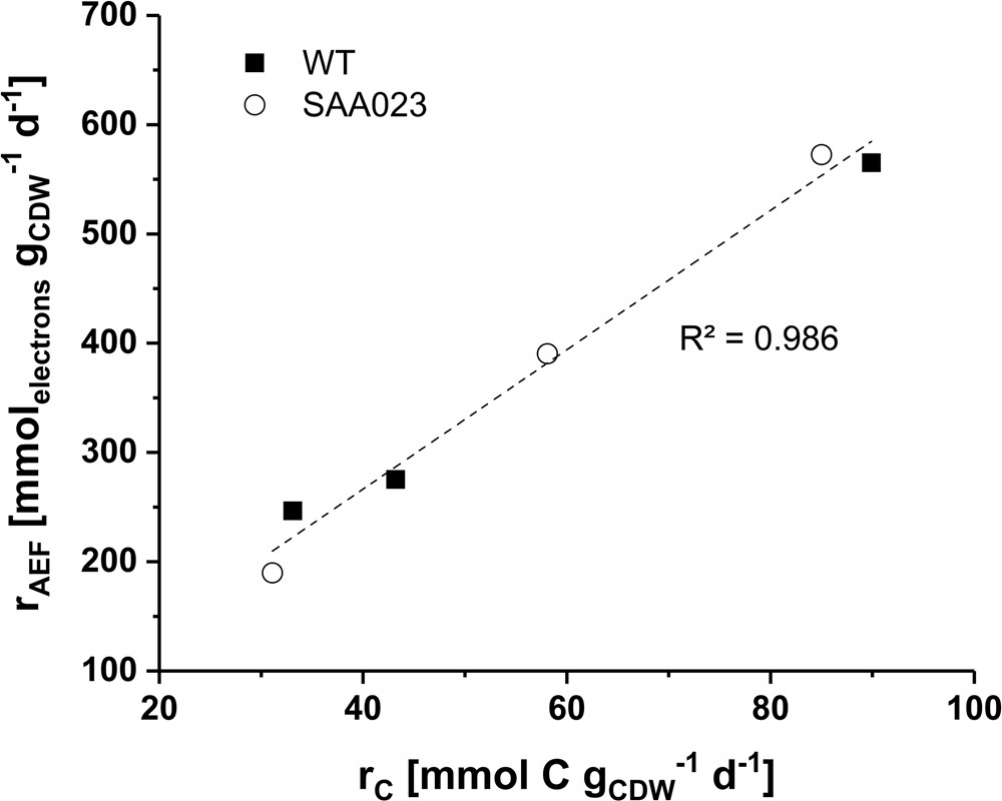
Correlation of total carbon production rates (*r_C_*) and alternative electron fluxes (*r*_AEF_) for *Synechocystis* WT and SAA023 under the different experimental conditions tested in continuously operated flat panel photobioreactors (see the legend to Fig. 2 for details). Linear fitting was performed with mean values of r_C_ and r_AEF_.

Interestingly, we found a strong correlation of carbon production rate and AEF under all cultivation conditions (Fig. 3). This indicates that the ATP demand increases with the carbon production rate and is covered by a higher AEF. However, it remains an open question of why WT cells under HLLC were not able to better adjust the AEF and thus better exploit their carbon assimilation capacity. A possible explanation relates to the activity of carbon concentrating mechanisms (CCMs). It is known that cyanobacteria possess five different energy-dependent uptake systems for inorganic carbon (C_i_). CCMs of *Synechocystis* typically are induced under carbon-limited conditions such as HLLC (47, 48). Although it was shown that the energy required for active C_i_ uptake depends on photosynthetic activity, it is still not clear if and to what extent there is a direct consumption of ATP by C_i_ transporters and/or a dependency on electrochemical gradients (48). Such energy dependence can be expected to rely on an activation of cyclic electron transport around PSI (47) and other AEFs. For the present study, it can be argued that WT cells under HLLC conditions cannot fully meet the energy requirement for both active C_i_ uptake and carbon fixation in the CBB cycle, as they cannot reach an appropriately high ATP/NADPH ratio. This imbalance may be at least partially counterbalanced by lactate production in SAA023 cells, with its lower ATP over NADPH demand compared to biomass formation, enabling a higher carbon fixation rate. The quantum requirement of carbon production (*Q_C_*) (Table 1) is proposed as a good indicator of the source/sink balance, thereby supporting the optimization of growth conditions.

### Conclusions.

**FIG 4:**
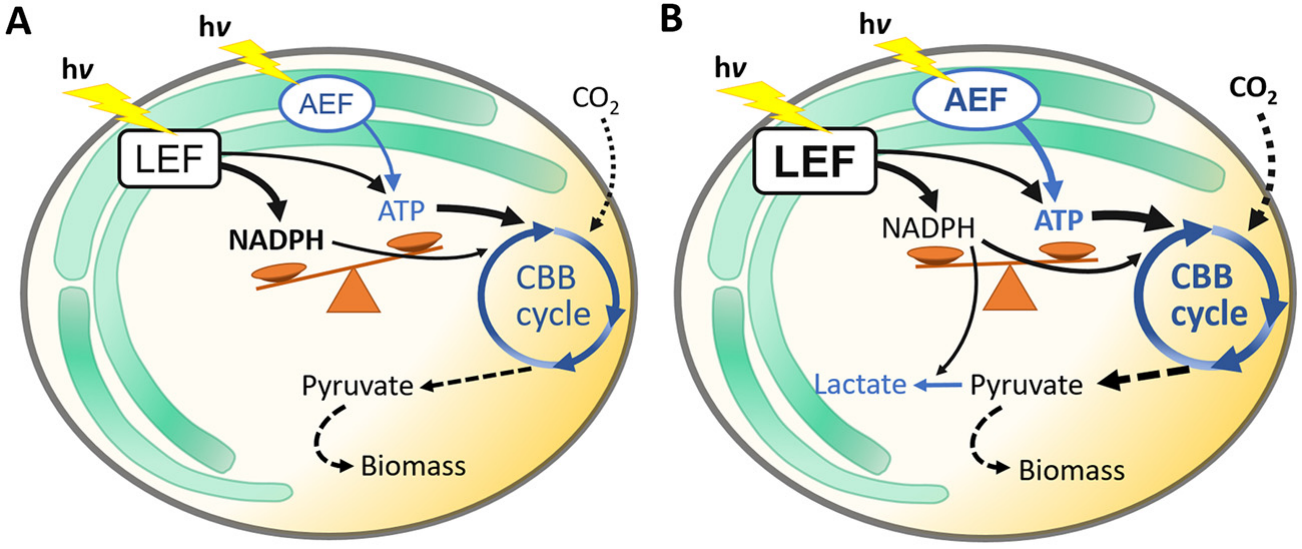
Schematic representation of source/sink relationships in *Synechocystis* WT (A) and strain SAA023 (B). Absorbed light and its conversion into photosynthetic electrons provides the “source” of metabolic energy in the form of NADPH and ATP, which fuel CO_2_ fixation in the Calvin-Benson-Bassham (CBB) cycle. Thereby, linear electron flux (LEF) provides both NADPH and ATP, whereas alternative electron flux (AEF) only provides ATP. In WT cells (A), the NADPH/ATP ratio provided by LEF does not meet the requirements of the CBB cycle (indicated by the thin arrow for ATP production by LEF) and can be compensated for by AEF, which, however, depends on the conditions and may result in saturation and depletion of NADPH and ATP pools, respectively (indicated by the imbalance of NADPH and ATP). In consequence, the cells are forced to downregulate their photosynthetic activity and are not able to operate at their maximum capacity. (B) The introduction of NADPH-consuming heterologous carbon sinks (i.e., lactate in the present study) releases unused potential, not only regarding the light reaction (indicated by “LEF” in boldface), but also with respect to carbon fixation (indicated by “CBB cycle” in boldface and larger arrows), which is known as the “carbon sink effect.” Thereby, heterologous carbon sinks can support the adjustment of the NADPH/ATP ratio and thus sink/source balancing. The present study shows that an explicit carbon sink effect is observed only in cells dealing with a sink limitation and that light and CO_2_ supply can be optimized so that WT cells also achieve a well-balanced metabolic state. Under such optimized conditions, the extent of the carbon sink effect is strongly diminished.

The present study reports the establishment of defined metabolic source/sink ratios in continuous cultures to investigate the carbon sink effect often observed in *Synechocystis*. The introduction of an NADPH-consuming heterologous carbon sink (i.e., lactate formation) increased the photosynthetic efficiency under all growth conditions investigated. An increased overall carbon fixation rate in response to the heterologous carbon sink only was observed for cells growing at a high source/sink ratio (i.e., under carbon limitation, as typically encountered during the day under atmospheric conditions). This carbon sink effect appears to be induced by an optimized ATP/NADPH balance in lactate-producing cells (summarized in Fig. 4), with *Q_C_* constituting an excellent indicator of the source/sink balance and thus a key parameter for photobioprocess optimization. The strong correlation found for carbon fixation rate and AEF indicates that the primary role of AEF in *Synechocystis* is the provision of additional ATP for carbon fixation instead of dissipating excessively absorbed light energy. The most important prerequisites for a high product yield (in this case lactate) in *Synechocystis* are a sufficient light supply (source capacity) together with a high proportion of AEF. In contrast, the supply of inorganic carbon appears subordinated.

## MATERIALS AND METHODS

### Bacterial strains.

*Synechocystis* sp. strain PCC 6803 (originating from the Bhaya Lab, Stanford) and the lactate-producing strain SAA023, harboring a lactate dehydrogenase gene, were provided by K. J. Hellingwerf and F. Branco Dos Santos from the University of Amsterdam (see details in references 13 and 49). All strains were maintained as cryo-cultures at −80°C in BG11 medium supplemented with 20% dimethyl sulfoxide (DMSO) and freshly cultivated prior to experiments.

### Growth conditions.

*Synechocystis* strains were cultivated as described before (22). In short, BG11 (50) supplemented with 1.5% (wt/vol) agar and 0.03% (wt/vol) sodium thiosulfate was used for agar plate cultivation and YBG11 (51) for liquid cultivation. YBG11 was supplemented with 50 mM HEPES for shaking flask cultivations and 2 mM HEPES for bioreactor cultivations. The medium was adjusted with 10 M NaOH to a pH of 8.0. The standard cultivation conditions were 20 μmol photons m^−2^ s^−1^, 30°C, and 75% relative humidity (rH) in a plate incubator (poly klima GmbH, Freising, Germany) for agar plates and 140 rpm, 30°C, ambient CO_2_ concentration (0.04%), 50 μmol photons m^−2^ s^−1^, and 75% rH for shaking flask experiments (batch cultivation) and for bioreactor precultures (Multitron; Infors AG, Bottmingen, Switzerland).

Continuous cultivations were carried out in flat-panel airlift bioreactors (Labfors 5 Lux; Infors AG, Bottmingen, Switzerland) as described before (22). Standard conditions involved pH 8.0 (controlled with HCl and NaOH during all experiments), 30°C, and aeration at 1.0 vessel volume per minute (vvm) (pressurized air or synthetic air enriched with CO_2_ when appropriate). Illumination was provided by white light LEDs (see Fig. S1 in the supplemental material). Three different conditions were tested: (i) light limitation (low light intensity of 65 μmol photons m^−2^ s^−1^ and aeration with 1% CO_2_ [LLHC]), (ii) excess supply (high light intensity of 250 μmol photons m^−2^ s^−1^ and aeration with 1% CO_2_ [HLHC]), and (iii) carbon limitation (high light intensity of 250 μmol photons m^−2^ s^−1^ and aeration with approximately 0.05% CO_2_, considered ambient [HLLC]). The given intensity of irradiance represents the incident light intensity in the cultivation vessel, measured with a ULM-500 equipped with a spherical 360° sensor (US-SQS/L; Heinz Walz GmbH, Effeltrich, Germany). The biomass concentration was kept constant by feeding fresh medium via a peristaltic pump (IPC series, ISMATEC; Cole-Parmer GmbH, Wertheim, Germany) into the bioreactor at a rate equal to the growth rate under the respective conditions (cultivation in turbidostat mode). The reactor filling level was kept constant by pumping culture through a fixed efflux tube at the top of the reactors. The growth rate was calculated from the dilution rate, *D*, according to $D=\mu =\frac{\dot{V}}{V}$, with a working volume (*V*) of 1.8 L and the feeding rate $\dot{V}$(L day^−1^). For each growth condition, a new turbidostat bioreactor was started by inoculation from a batch preculture. The described parameters (see below) were measured on three different sampling days for each growth condition, with at least one complete exchange of the culture volume in the bioreactor in between the sampling days.

### Determination of cell dry weight, C/N ratio in biomass, and Chl*a* content.

The cell dry weight (CDW), C/N content of biomass, and Chl*a* content were determined as described before (22). CDW and Chl*a* content were determined in triplicates, the C/N ratios in duplicates for each measurement day (3 days per condition and strain). OD_750_ and cell number were measured at least four times per day as described before (22, 52).

### Determination of carbon fixation rate and carbon partitioning.

The carbon production rate was derived from the biomass formation rate and the relative carbon content of biomass. Biomass concentration was analyzed by centrifugation of a 50-mL cell suspension (centrifuge 5810R, rotor FA-45-6-30; Eppendorf AG, Hamburg, Germany) in a glass tube for 10 min at 10°C and 7,000 × *g*. The supernatant was discarded, and the pellet was washed with distilled water. The cells were dried at 70°C until a constant weight was reached. Biomass composition was evaluated on the elemental level.

The C/N ratio was analyzed by centrifuging 50-mL cell suspension for 10 min at 10°C and 7,000 × *g* (centrifuge 5810R; rotor FA-45-6-30, Eppendorf AG, Hamburg, Germany). The supernatant was discarded, and the pellet was washed with distilled water. The samples were lyophilized (Freezone 2.5; Labconco, Kansas City, MO, USA) and stored until further measurement. The measurement of the carbon and nitrogen content of the dried biomass was performed with a Vario EL Cube elemental analyzer (Elementar Analysegeräte GmbH, Langenselbold, Germany).

Initially, the biomass formation rate, *r_X_*, (g_CDW_ L^−1^ day^−1^) was calculated with the equation *r_x_* = *D* × *X*, with *D* (dilution rate) = growth rate day^−1^ and *X* representing the biomass concentration in g_CDW_ L^−1^.

The growth-related carbon production rate, *r_C,X_* (mmol C g_CDW_^−1^ day^−1^), was then derived from *r_X_*, and the relative carbon content of dry biomass (*C*) was derived from C/N measurements:

$$r_{C,X}=\frac{r_{X}\times C}{\text{CDW}}$$

The total carbon production rate, *r_C_* (mmol C g_CDW_^−1^ day^−1^), is calculated as the sum of *r_C,X_*, and the carbon-based lactate formation rate, *r*_Lac_ (mmol C g_CDW_^−1^ day^−1^), is calculated by *r_C_* = *r_C,X_* + *r*_Lac_. Biomass and lactate formation rates were calculated from values of three independent measurement days.

### Quantification of lactate and lactate production rate.

Lactate was quantified in the supernatant of the cell suspension. Samples of cell suspension of 0.5 to 1.0 mL were centrifuged for 5 min at 17,000 × *g* and 4°C (Heraeus Fresco 17; Thermo Scientific, Waltham, MA, USA). The supernatant was stored at −20°C until further analysis. The lactate concentration was determined either by high-performance liquid chromatography (HPLC) (Ultimate 3000 series; Dionex, Thermo Scientific) equipped with a refractive index (RI) detector (Refractomax 520; Thermo Scientific) or an assay kit according to the supplier’s instructions (MAK065; Sigma-Aldrich). For quantification via HPLC, a HyperREZ XP carbohydrate H^+^ column was used with 5% sulfuric acid as mobile phase at 40°C and a flow rate of 1.2 mL min^−1^. The specific lactate production rate (mmol g_CDW_^−1^ day^−1^) was calculated based on the lactate concentration in the supernatant (*c*_Lac_) (mmol L^−1^), the biomass concentration (CDW in g L^−1^), and the dilution rate (*D*) (day^−1^) with the equation

$$r_{\text{Lac}}=\frac{c_{\text{Lac}}\times D}{\text{CDW}}$$

For the calculation of carbon partitioning into lactate, *r*_Lac_ was converted into a carbon-based production rate (mmol C g_CDW_^−1^ day^−1^).

### Quantum requirement of carbon production.

In the present study, the quantum requirement per fixed carbon (*Q_C_*) (mol quanta mol C^−1^) describes the efficiency of the usage of absorbed light energy for total carbon production (including heterologous products). When cells are forced to dissipate absorbed light nonphotochemically (46), this dissipated light energy is not available anymore for carbon assimilation and results in a high quantum requirement. *Q_C_* was calculated with the equation

$$Q_{C}=\frac{Q_{\text{phar}}}{r_{C}}$$

where *Q*_phar_ is the amount of absorbed photosynthetically available radiation (mol quanta g_CDW_^−1^ day^−1^ [see below]).

### Measurement of the effective quantum yield at PSII.

The effective quantum yield at PSII, Y(PSII), was measured and calculated as described before (22) using a Multi-Color PAM (MC-PAM) device (53) (Heinz Walz GmbH, Effeltrich, Germany). The wavelength of the measurement light was set to 400 nm in order to minimize underestimation of Y(PSII) due to excitation of phycobilisomes at higher wavelengths (54, 55) (see Fig. S2 in the supplemental material). In short, a sample from the bioreactor was instantly transferred into the measuring cuvette of the MC-PAM to determine Y(PSII) in the light-acclimated state of the cells. In order to create highly comparable conditions in the measuring cuvette as in the bioreactor, white actinic light with increasing light intensity was applied for the light induction curve (LIC). Analysis was conducted with undiluted samples, which was possible due to the low biomass concentration in the photobioreactor. The measurements were conducted at least nine times per steady state (minimum three times per measurement day on three independent measurement days).

### Measurement of photosynthetic rates.

The following photosynthetic parameters were determined as described previously (22, 56–59): the Chl*a*-specific absorption coefficient of the cells (a*_phy_), the cellular quantum absorption rate (*Q*_phar_), the net O_2_ evolution rate (*r_O_*), and the maximum electron flux at PSII (*r_F_*).

*Q*_phar_ was determined as described by Gilbert et al. (59) according to the equation

$$Q_{\text{phar}}=\overset{\text{700 nm}}{\underset{\text{400 nm}}{\int }}Q\left(\text{λ}\right)-Q\left(\text{λ}\right)\times e^{\left(-a^{\text{*}}(\text{λ})\times \text{Chl}a\times d\right)}$$

with *Q*(λ) representing wavelength-dependent incident radiation (μmol m^−2 ^nm^−1^ s^−1^), a* representing the wavelength-dependent Chl*a*-specific absorption coefficient [m^2^ mg Chl*a*^−1^], Chl*a* representing the chlorophyll *a* concentration (mg Chl*a* m^−3^), and *d* representing the optical path length (m).

To determine *Q*_phar_, the emission spectrum of the LED panel of the reactor (Fig. S1) and the specific *in vivo* Chl*a* absorption spectrum of the cells have to be measured (56, 59). The absorption spectra were measured with a dual-beam spectrophotometer (Zeiss M500; Carl Zeiss AG, Oberkochen, Germany). The emission spectra of the light sources were determined using a spectroradiometer (Tristan 4.0, m-u-t GmbH, Wedel, Germany).

The net O_2_ evolution rate, *r_O_*, was measured in triplicates on three different days under the respective conditions using a Clark-type electrode (MI-730; Microelectrodes, Inc., Bedford, NH, USA). An undiluted sample was transferred into a measurement cuvette and illuminated with white actinic light with increasing light intensities; each light period was followed by a dark phase. This ensured that the conditions in the reactor and during the assay were as comparable as possible. O_2_ evolution rates were determined for all light intensities and the respective respiration rates in a following dark phase (see also reference 60). The gross O_2_ formation rate was calculated by correcting the measured O_2_ evolution rate for the corresponding respiration rate. The maximum electron transport rate at PSII (*r_F_* [expressed as an O_2_ evolution rate]) was estimated based on the measured Y(PSII) according to reference 59 with the equation

$$r_{F}=\frac{\text{Y}\left(\text{PSII}\right)\times Q_{\text{phar}}\times \text{Chl}a_{\text{CDW}}\times 0.5\times 0.25}{d\times c\left(\text{Chl}a\right)}$$

where *d* (m) is the diameter of the bioreactor vessel, Chl*a*_CDW_ is the specific chlorophyll *a* content (mg g_CDW_^−1^), and *c*(Chl*a*) is the volumetric Chl*a* concentration (mg m^−3^). The factors 0.5 and 0.25 account for the assumption that two quanta are required for the transfer of one electron into the photosynthetic electron transport chain and that four electrons are required per molecule O_2_ evolved, respectively. The factor 0.5 also assumes an equal distribution of absorbed light onto both photosystems, PSII and PSI. Thus, *r_F_* represents the estimated total electron transport rate at PSII not considering AEF (61). Consequently, the amount of AEF can be estimated by the equation *r*_AEF_ = *r_F_* − *r_O_*, where the measured gross oxygen evolution is affected by AEF: e.g., cyclic electron flow at PSI or electron transfer to O_2_ by the Mehler reaction (37). We are aware that the estimation of *r_F_* in cyanobacteria can be hampered by phycobilisome-related fluorescence emission (54, 55). However, these effects were minimized by the careful choice of the wavelength for measuring chlorophyll fluorescence (see above). This is supported by the fact that *r_F_* showed a strong correlation with *r_C_* (see Fig. S3 in the supplemental material), indicating that the estimated electron transport rate was not the subject of a systematic inadequacy of the applied fluorescence method. Photosynthetic rates were determined on three independent measurement days. *r_O_* and *r_F_* are given as O_2_ evolution rates in mmol O_2_ g_CDW_^−1^ day^−1^. *Q*_phar_ is given as quantum uptake rate in mol photons g_CDW_^−1^ day^−1^.
