# Supplementary material for: Heterologous Lactate Synthesis in Synechocystis sp. Strain PCC 6803 Causes a Growth Condition-Dependent Carbon Sink Effect
Source: Appl Environ Microbiol. 2022 Apr 4;88(8):e00063-22. doi: 10.1128/aem.00063-22 (PMC9040622; doi:10.1128/aem.00063-22)
Supplement: Supplemental file 1 — Fig. S1 to S3. Download aem.00063-22-s0001.pdf, PDF file, 0.1 MB [file aem.00063-22-s0001.pdf]

Supplemental material

Heterologous lactate synthesis in Synechocystis sp. PCC 6803 causes a growth condition-dependent carbon sink effect

Marcel Grund, Torsten Jakob, Jörg Toepel, Andreas Schmid, Christian Wilhelm, Bruno Bühler

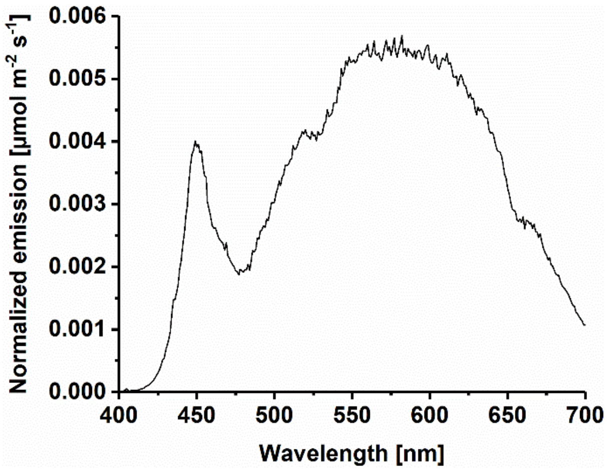

Fig. S1: Normalized emission spectrum of the bioreactor LED panel used for continuous cultivation of *Synechocystis* strains.

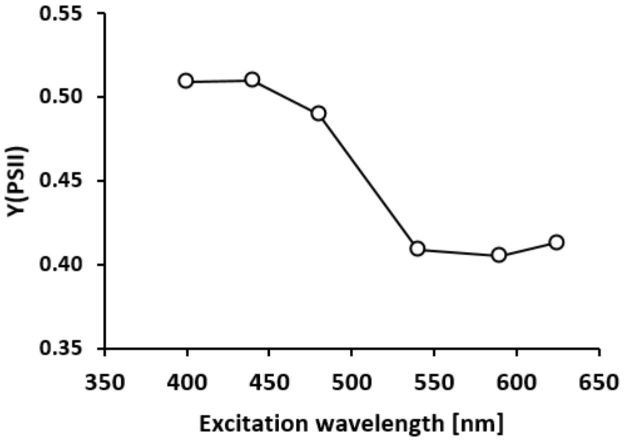

Fig. S2: Exemplary data of the quantum yield at Photosystem II (Y(PSII)) in *Synechocystis* sp. PCC6803 measured at different wavelength of the measuring light.

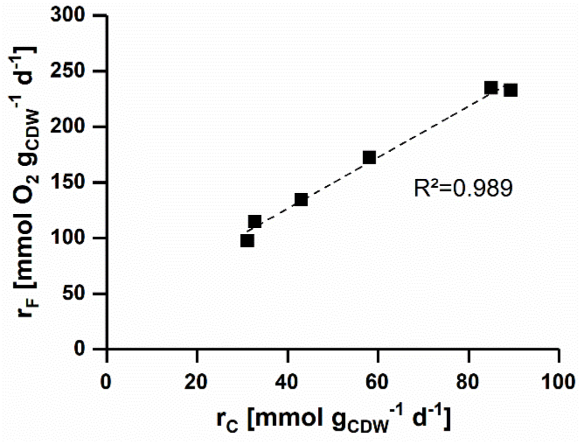

Fig. S3: Correlation of the carbon biomass production rate (r_C) with the fluorescence-based electron transport rate at Photosystem II (r_F; converted into oxygen evolution rate) in *Synechocystis* sp. PCC6803 under continuous cultivation with different source/sink conditions.
